# PPESK-Modified Multi-Functional Epoxy Resin and Its Application to the Pultrusion of Carbon Fiber

**DOI:** 10.3390/polym10101067

**Published:** 2018-09-26

**Authors:** Liwei Wang, Jinyan Wang, Fengfeng Zhang, Yu Qi, Zhihuan Weng, Xigao Jian

**Affiliations:** 1 Liaoning High Performance Resin Engineering Research Center, Department of Polymer Science and Materials, Dalian University of Technology, Dalian 116024, China; jhwlw2008@163.com (L.W.); wangjinyan@dlut.edu.cn (J.W.); Zhangfeng0908@126.com (F.Z.); qiyudy001@mail.dlut.edu.cn (Y.Q.); 2 PetroChina Co., Ltd., Jilin Petrochemical Branch, Jilin 132022, China

**Keywords:** carbon fiber, multi-functional epoxy resin, polyethersulfone resin, pultrusion process, curing

## Abstract

Multi-functional epoxy resins are generally brittle due to their high crosslinking densities, which can limit their use for applications that require impact resistance. Pultruded poly(phthalazinone ether sulfone ketone) (PPESK)-modified epoxy resins were prepared and their curing behaviors, heat resistance properties, and viscosity changes investigated. The glass transition temperature of these resins was found to increase with increasing PPESK content; however, these values were still compatible with the pultrusion process. Little change in the tensile strength and elongation lengths at breaking point were observed for blended PPESK/multi-functional epoxy resin containing 4–6% PPESK, and its viscosity levels were still within the requirements of the pultrusion process. Carbon fiber/multi-functional epoxy resin/PPESK (CF/E/PPESK) composites were also prepared and their performance investigated. The bending radius of these PPSEK-modified composites could reach up to 55 D with no cracking or peeling observed in their surface layers. The fatigue frequency of the sinusoidal waveforms for the composite did not change after one million fatigue test cycles, meaning that a strength retention rate of >90% was achieved. Therefore, this study describes a powerful approach for preparing toughened multi-functional epoxy resins that are well suited to pultrusion processes.

## 1. Introduction

The advantages of using multi-functional epoxy resins include high temperature resistance, low shrinkage, good stability, and high mechanical strength. However, the brittle nature of these resins after curing, their poor resistance to impact, and the appearance of stress cracks over time limits their application under harsh conditions [1,2,3]. Modified multi-functional epoxy resins have been widely studied in recent years, resulting in tough high-temperature epoxy resins now being available [4,5,6]. Many methods have also been developed to successfully enhance the fracture toughness of epoxy resin [7,8,9], with rubbers often used as effective toughening additives for epoxy resin. However, the presence of nanorubber particles dispersed throughout the resin matrix can result in a large increase in its viscosity, which can cause difficulties when it is used for pultrusion.

Another powerful alternative for toughening the epoxy resin is to use thermoplastics, which can maintain both the mechanical and thermal properties of the resin. These toughened epoxy resins are produced through blending epoxy resin with high-performance engineering thermoplastics, such as polysulfone [10], poly(ether sulfone) [8,11], poly(ether imide) [12,13], poly(amide-amidic acid) [14] and poly(ether ketone) [15,16]. Reaction-induced phase separation is the major toughening mechanism currently used to engineer thermoplastic/epoxy blend composites [12,17]. We have previously reported a series of engineering phthalazinone-derived thermoplastics, whose twisted aromatic structures confer high mechanical strengths and good thermal properties on the toughened epoxy resins [18,19,20,21]. For example, a novel thermoplastic poly(phthalazinone ether nitrile ketone) (PPENK) with a *T*_g_ of 280 °C [22] was blended with an epoxy-based tetraglycidyl 4,4′-diaminodiphenyl-methane (TGDDM)/bisphenol A-type novolac (F-51) resin that was cured using 4,4′-diaminidiphenysulfone (DDS) [20]. This resulted in the formation of a composite with numerous intermolecular and intramolecular hydrogen cross-links throughout the TGDDM/F-15/PPENK blend. These hydrogen bonding interactions resulted in good compatibility between the PPENK and the epoxy resin, with the composite’s critical stress intensity factor and impact strength values reaching a maximum when 10 phr PPENK was used.

Poly(phthalazinone ether sulfone ketone) (PPESK) is a new type of high-performance, temperature-resistant resin characterized by its resistance to high temperatures and a good solubility profile, with a glass transition temperature of between 250–310 °C. The good toughness, high modulus, heat resistance, and excellent overall performance of PPESK resins makes them suitable as modifiers for strengthening multi-functional epoxy resins [2]. PPESK-modified multi-functional epoxy resins could be potentially used for pultrusion of high-temperature composites that should exhibit good temperature resistance, improved resistance to impact, and reduced stress cracking.

In this study, the curing behavior, compatibility, viscosity change, and related mechanical properties of CF/PPESK-modified multi-functional epoxy resins were investigated. These resins were found to exhibit good mechanical and torsional strength properties, excellent resistance to fatigue, and good aging properties.

## 2. Materials and Methods

### 2.1. Materials

Polyphenylene ether sulfone resins (PPESK) with an average molecular weight of 4.52 × 10^4^ g/mol were purchased from Dalian Polymer New Materials Co. Ltd. (Dalian, China). Carbon fibers (CF, JHTD-45, 12 K) with a tensile strength of 4.9 GPa were obtained from the Carbon Fiber Factory of China Petroleum Jilin Petrochemical Company (Jilin, China). Multi-functional epoxy resins (CP02A) and an acid anhydride curing agent (CP02B) were purchased from Changshu Jiafa Resin Co. Ltd. (Changshu, China). Reagent-grade chloroform (99%) was obtained from Shanghai Youshi Chemical Co. Ltd. (Shanghai, China). A release agent (1890 M) was purchased from Beijing Kesila Technology Co. Ltd. (Beijing, China).

### 2.2. Preparation of Samples

Preparation of blended resins. Blended solutions were prepared using 0, 2, 4, 6, and 8 phr of PPESK in CP02A chloroform, where phr represented the weight of PPESK per hundred parts of CP02A. The solvent was then removed using vacuum evaporation at 70 °C. The resultant PPESK was then mixed with CP02B and 1890 M and magnetically stirred to produce a homogeneous solution. The blended resin was poured into a mold, vacuum defoamed, and cured at 90 °C/2 h, 160 °C/4 h and 200 °C/2 h sequentially. Samples were then cooled to room temperature and demolded. This general procedure was used to prepare blended resins containing different doses of PPESK of 0, 2, 4, 6, and 8 phr that were classified as E/PPESK-0, E/PPESK-2, E/PPESK-4, E/PPESK-6 and E/PPESK-8, respectively.

**Figure 1:**
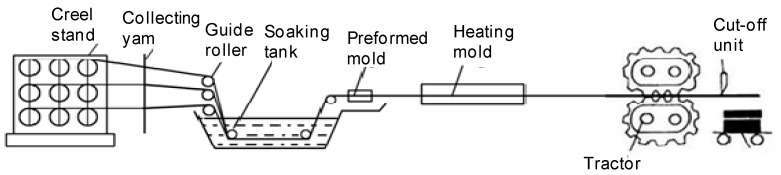
Carbon fiber bar pultrusion process.

Preparation of pultruded carbon fiber bars. An overview of the method used to produce the carbon fiber pultruded bars is shown in Figure 1 and Table 1. Different mass ratios of PPESK/CP02A, CP02B and 1890 M (internal release agent containing 1–2% CP02A) were stirred for 10–20 min. Each of the blended resin mixtures was then transferred to a dipping tank, and a pultrusion machine used to perform dipping and pultrusion. For example, a φ25 carbon fiber bar was prepared using 700 shafts of JHTD45-12K carbon fiber that was then passed through the dipping tank to create a preformed mold that was heated and then processed through a tractor and cutting machine. The resulting composites were classified as CF/E/PPESK.

### 2.3. Characterization

The thermal stabilities of the epoxy resins were tested using differential scanning calorimetry (DSC, Mettler Toledo, Zurich, Switzerland) and thermogravimetric analysis (TGA, Mettler Toledo, Zurich, Switzerland). For DSC analysis, the glass transition temperature of cured 10 mg samples was measured on a Mettler DSC1 differential scanning calorimeter, using a heating rate of 10 °C/min over a temperature range of 50–350 °C. Thermogravimetric analysis (TGA) was carried out using a Mettler TGA1 thermal gravimetric analysis instrument under a flow of nitrogen (50 mL/min) at a heating rate of 10 °C/min over a temperature range of 30–700 °C. The tensile strength of the composites was determined using a WSM-50KN electric universal testing machine (Intelligent Instrument Equipment, Changchun, China) according to the GB/T1447-2005 fiber-reinforced plastic experimental protocol, using a displacement rate of 2 mm/min for five repeat samples. Torsional strengths were determined according to the standard GB/T10128-2007 method using a NWS1000 microcomputer-controlled torsion test machine (Changchun Research Institute for Mechanical Science Co., Ltd., Changchun, China) operating in chuck angle control mode at a rate of 6°/min. Tensile fatigue tests were conducted on a SDS-200 electro-hydraulic-servo fatigue testing machine (Changchun Research Institute for Mechanical Science Co., Ltd., Changchun, China) at 25 °C using a 10 Hz frequency for one million cycles. This testing cycle involved a pull, release, press, release and pull cycle, using a pull force of 70 kN and a downward force of 10 kN. This mechanical process could be represented as a sine-wave, with its maximum wave peak corresponding to the maximum tensile force, and its lowest wave peak corresponding to the minimum pressure. Repetition of several cycles resulted in a decrease in the mechanical properties of the materials, with the resultant decrease in tensile force and pressure measured by software analysis of changes to the sine-wave curve. The morphology of the blended resin was determined by spraying the fracture surface of the resin with gold, followed by analysis using a KYKY2800B scanning electron microscope (SEM, KYKY Technology Co., Ltd., Beijing, China). Toughness tests were conducted according to the GB29324 standard by winding the composite resin onto a 55 D cylinder using a test rotation speed of 3 r/min.

## 3. Results

### 3.1. Curing Behavior and Viscosity of E/PPESK Resin Blends

**Figure 2:**
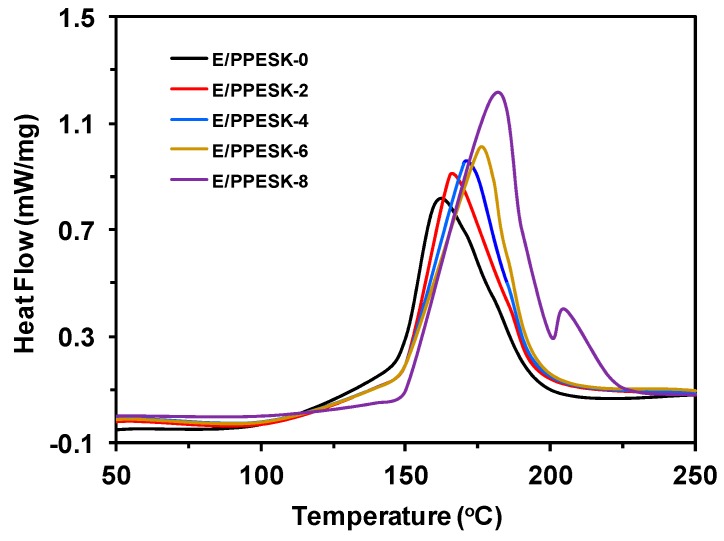
Dynamic differential scanning calorimetry (DSC) curves of resin systems at scanning rate of 10 °C/min.

Production of composite materials using pultrusion confers significant efficiencies associated with fast molding and high production rates, with the ideal process requiring low mixing viscosities, a slow increase in viscosity over time at room temperature, and a rapid heat-curing reaction. Consequently, the curing behavior of E/PPESK epoxy resin blends was examined to determine the impact of the PPESK content on the curing process. A step-up temperature program was used to investigate the curing behavior of composite epoxy resins, with the curing behavior of different PPESK mass ratios (0, 2, 4, 6, and 8 phr) determined using DSC at a heating rate of 10 °C/min (see Figure 2). The heat released from the curing reaction and the curing temperature were found to increase slightly as the PPESK content increased. This is because PPESK has a relatively high glass transition temperature (280 °C), with small amounts of PPESK serving to plasticize the network structure of the epoxy resin. This plasticization is beneficial to the overall flow of the epoxy resin, which results in an increase in the cross-linking density [23] and heat resistance of the polymer blend. The high glass transition temperature of PPESK means that distortion of the network structure of the epoxy resin chains becomes increasingly difficult as the PPESK content increases.

**Figure 3:**
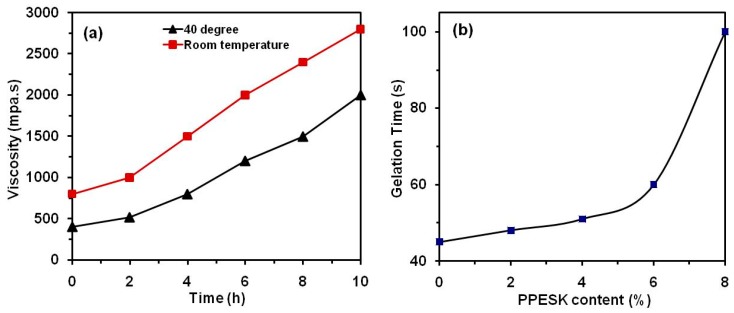
(**a**) viscosity against time curves at ambient temperature and 40 °C; (**b**) gelation time against PPESK content curve.

Epoxy resins that exhibit high temperature resistance normally have a relatively high room-temperature viscosity. The viscosity of epoxy resins can be controlled by heating during processing, which enables good fluidity to be maintained and thus improves infiltration of the resin into the carbon fibers. An effective pultrusion process requires the blended resin to be stable at temperatures below 40 °C. The best viscosity stability levels we observed were for blended E/PPESK resins containing 6 phr PPESK over a 6 h period (see Figure 3a). It was found that addition of PPESK had little effect on the viscosity of the resin, which was also relatively stable at higher temperatures; this is likely to be due to no steric hindrance being present. However, the gelation time of the system was found to increase significantly when the content of PPESK exceeded 6% (Figure 3b), which was detrimental to the pultrusion process and also affected the curing behavior of the resin.

The heat resistance of pultruded epoxy resins is usually investigated by measuring their glass transition temperatures, with higher glass transition temperatures normally affording pultruded resins with greater thermal stabilities at higher temperatures. The glass transition temperatures of the blended resins were found to increase with increasing PPESK content (see Table 2), with TGA measurements showing that their initial thermal decomposition and maximum weight loss temperatures were essentially unchanged. The blended resins remained intact below temperatures of 385 °C, indicating that addition of PPESK did not adversely affect the overall thermal stability of the epoxy resins.

### 3.2. Mechanical Properties of E/PPESK Resin Blends

**Figure 4:**
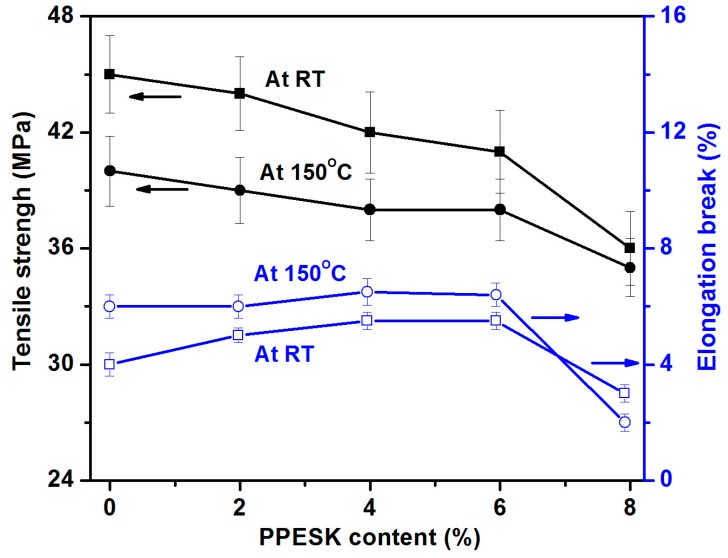
The effect of PPESK content on tensile strength and elongation at break.

**Figure 5:**
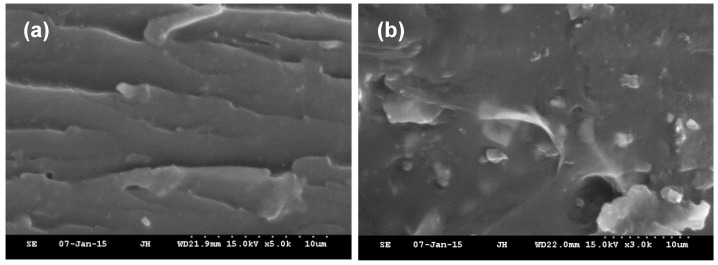
Scanning electron microscope (SEM) micrographs of the fractured surface of failed specimens for epoxies modified with (**a**) 6% and (**b**) 8% PPESK.

Little change in the tensile strength and elongation length at breaking point was observed for the blended resins that contained 4–6% PPESK content, which reflects the good physical compatibility between these two components (see Figure 4). In contrast, the mechanical properties of the system changed significantly when the content of PPESK exceeded 6%. SEM images of E/PPESK epoxy resins containing varying PPESK content revealed obvious separation between the two phases of the resin when the PPESK content was 8% (see Figure 5). It is known that poor compatibility between the two phases of a resin used for pultrusion results in the binding forces of their composite materials being decreased dramatically. Consequently, the physical properties of pultruded CF/E/PPESK epoxy resins containing ≥8% PPESK dosage were not investigated in this study.

### 3.3. Physical Properties of CF/E/PPESK Epoxy Resin Composites

**Figure 6:**
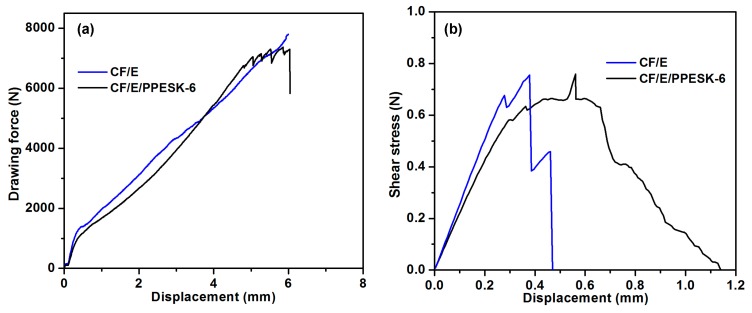
Tensile strength (**a**) and interlaminar shear strength (**b**) of carbon fiber/multi-functional epoxy resin (CF/E) and CF/E/PPESK-6.

**Figure 7:**
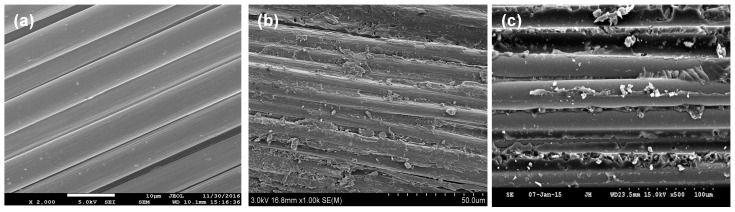
(**a**) SEM micrograph of the surface of carbon fiber (CF-12K); (**b**) CF/E epoxy resin composite; and (**c**) CF/E/PPESK-6 epoxy resin composite.

**Figure 8:**
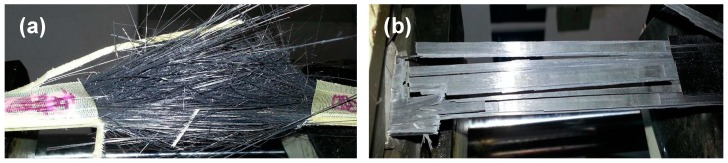
Pictures of fractured composites: (**a**) CF/E epoxy resin composite; and (**b**) CF/E/PPESK-6 epoxy resin composite.

The tensile strength and interlaminar shear strength of CF/E/PPESK-6 epoxy resin composites were found to decrease slightly (see Figure 6 and Table 3), with only a small decrease in the binding of the resin to the carbon fibers being observed after addition of PPESK. The surface morphologies of the carbon fibers and the composite are shown in Figure 7, while images of fractured composites that were produced in stress tests are shown in Figure 8. The composite fractures are filamentous, with their clusters exhibiting a divergent-like appearance. However, fractures became flaky and loosely distributed when the PPESK content was too high or too low, resulting in a non-uniform distribution throughout the resin. Modification of the resin with 6% PPESK resulted in the bending performance of the composite being significantly improved, enabling a value of 55 D to be achieved without any cracking or peeling of its surface layer occurring. This level of bending performance is highly desirable, because continuous pultruded composite materials are often incorporated into coiled materials.

### 3.4. Mechanical Properties of CF/E/PPESK Epoxy Resin Composites

**Figure 9:**
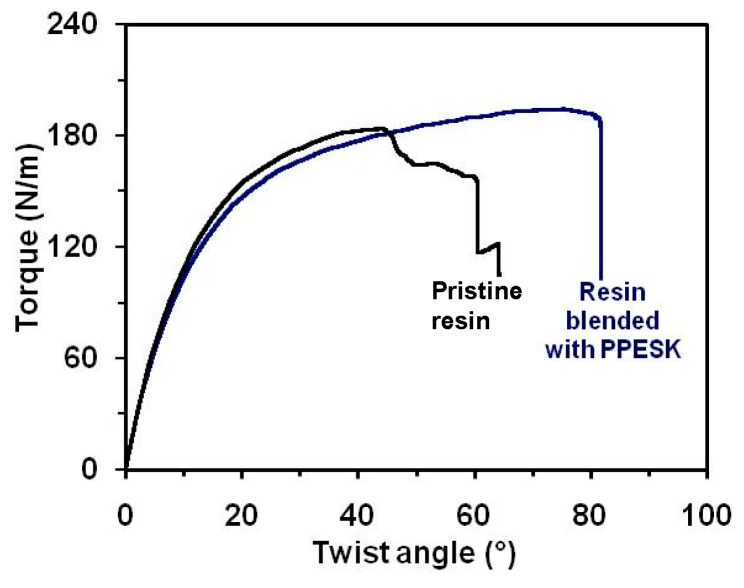
Torsion strength test on pristine resin (CF/E) and resin blended with PPESK (CF/E/PPESK-6).

Torsion strength tests revealed no obvious change in torque for the PPESK-modified resin CF/E/PPESK-6 when compared to unmodified multi-functional epoxy resin CF/E within a 10° twist range (see Figure 9). Unmodified epoxy resin was found to lengthen and fracture as the torque angle exceeded 10°; however, the torque applied to the PPESK-modified composite could be increased to >80° before it finally fractured. Therefore, the tensile strength of the PPESK composites was much greater than that of multi-functional epoxy resin, with both materials fracturing suddenly when their torque limits were exceeded.

### 3.5. Fatigue and Aging Properties of CF/E/PPESK Composites

**Figure 10:**
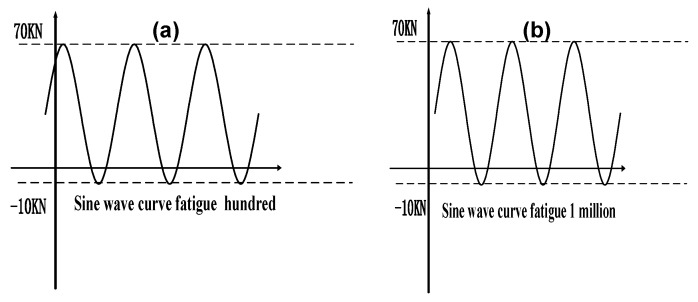
Fatigue sinusoids (**a**) before and (**b**) after one million cycles.

A million fatigue test cycles were conducted on the CF/E/PPESK-6 composite using a testing frequency of 10 Hz, a pull force of 70 kN, and a downward force of 10 kN. Structural displacement of the composite was found to be less than 1% after a million cycles (see Table 4), with no obvious changes in its waveform frequency being detected (see Figure 10). The strength retention rate of a CF/E/PPESK-6 composite after the fatigue tests had been completed was found to be >90% of its original value.

**Figure 11:**
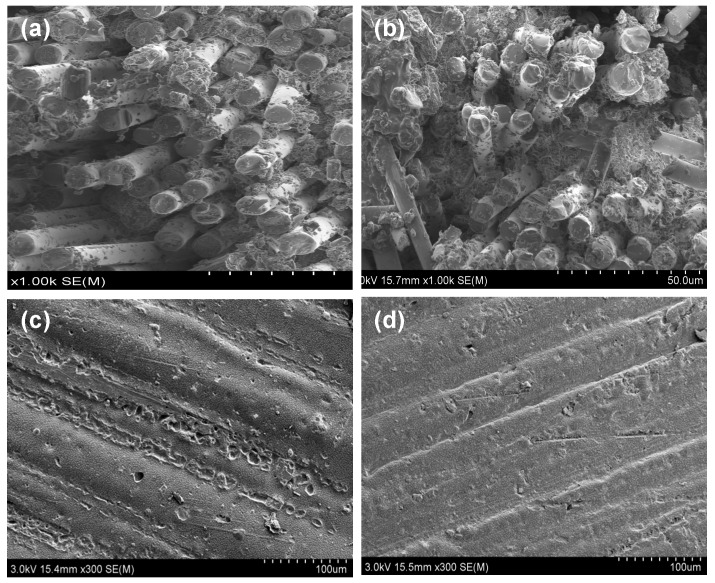
Microstructure of the sample CF/E/PPESK (**a**) before and (**b**) after 50 days of hydrothermal aging, as well as (**c**) before and (**d**) after 2160 h of ultraviolet aging.

The thermal, humidity (temperature 40 °C, humidity 60%, time 1200 h) and ultraviolet (wavelength 340 nm, temperature 50 °C, time 2160 h) aging properties of the CF/E/PPESK composites were subsequently determined. These studies revealed that the microstructure of the composite was affected by the tests, with composites experiencing some aging when treated with alternating high/low temperatures, high levels of moisture, and ultraviolet light over extended periods of time (see Figure 11). However, no damage leading to excessive cracking or oxidative corrosion (or other chemical reactions) was observed, with static tension tests revealing that the composites still retained 90% of their mechanical strength. This indicates that these composite materials exhibit good anti-aging and high corrosion resistance properties that should make them suitable for challenging polymer applications in relatively harsh environments.

## 4. Conclusions

This study has shown that poly(phthalazinone ether sulfone ketone) (PPESK) can be used to improve the heat resistance of multi-functional epoxy resins, increasing their glass transition temperature by 5 °C, which enables them to be used as resins for pultrusion. Good bonding was observed between the two components in E/PPESK epoxy resins containing 4–6% PPESK, which meant the resins’ tensile strengths and elongation lengths at breaking point were essentially unchanged. The mixed viscosity stabilities of these modified resins proved well suited to both the pultrusion and molding processes. The bending performance of composite bars prepared from the modified epoxy resins was improved significantly, with no cracking or peeling being observed in their surface layer for a bending radius of 55 D. Fatigue tests (1 million cycles) revealed that the CF/E/PPESK epoxy resin could maintain its shape effectively through multiple expansions and contractions, while aging tests (heat, temperature, humidity, and ultraviolet) resulted in an aged sample that retained 90% of its original strength.

## Figures and Tables

**Table 1:** Pultrusion conditions for carbon fiber/poly(phthalazinone ether sulfone ketone (CF/PPESK) resin/multi-functional epoxy resin composites.

Phase 1 (°C)	Phase 2 (°C)	Phase 3 (°C)	Mold Length (m)	Pultrusion Rate (m/min)	Resin Content (%)
170	180	195	0.9–1.2	0.4–0.6	20–24

**Table 2:** Effect of PPESK content on thermal properties of epoxy resin.

Samples	Glass Transition Temperature (°C)	Initial Decomposition Temperature (°C)	Maximum Weight Loss Temperature (°C)
E/PPESK-0	225	385	420
E/PPESK-2	226	386	421
E/PPESK-4	230	387	425
E/PPESK-6	232	385	417
E/PPESK-8	235	386	421

**Table 3:** Physical properties of CF/E/PPESK before and after toughening.

Properties	CF/E	CF/E/PPESK-6
Tensile strength (MPa)	2300 ± 142	2200 ± 153
Linear expansion coefficient	≤2.0 × 10^−6^	≤2.0 × 10^−6^
Interlaminar shear strength (MPa)	82.7 ± 4.8	81.6 ± 4.2
30 KN radial pressure resistance	No cracking or peeling	No cracking or peeling
Bending performance, 55D (D: bar diameter)	No cracking or peeling on the surface below 55D	No cracking or peeling on the surface at 55 D
High temperature tensile strength	Tensile strength at 190 °C ≥ the value of 90% at room temperature	Tensile strength at 190 °C ≥ the value of 90% at room temperature

**Table 4:** Parameters during the fatigue test for the composite CF/E/PPESK-6.

Cycles	Pull Force (kN)	Downward Force (kN)	Upward Displacement (mm)	Downward Displacement (mm)
3183	70.034	−9.961	0.942	0.134
6410	70.025	−10.044	0.96	0.131
142,036	70.025	−10.044	0.96	0.131
513,257	69.425	−10.044	0.98	0.132
784,601	69.925	−9.844	0.94	0.139
1,025,732	70.0855	−10.144	0.97	0.142
